# Interspecific coprophagia by wild red foxes: DNA metabarcoding reveals a potentially widespread form of commensalism among animals

**DOI:** 10.1002/ece3.9029

**Published:** 2022-07-03

**Authors:** Cristian N. Waggershauser, Pierre Taberlet, Eric Coissac, Kenny Kortland, Catherine Hambly, Xavier Lambin

**Affiliations:** ^1^ School of Biological Sciences University of Aberdeen Aberdeen UK; ^2^ Laboratoire d'Ecologie Alpine, CNRS Université Grenoble Alpes Saint‐Martin‐d'Heres France; ^3^ Forestry and Land Scotland Inverness UK; ^4^ Cairngorms Connect, Achantoul Aviemore UK

**Keywords:** commensalism, domestic dog, interspecific coprophagia, red fox, species interactions

## Abstract

Vertebrate animals are known to consume other species' faeces, yet the role of such coprophagy in species dynamics remains unknown, not least due to the methodological challenges of documenting it. In a large‐scale metabarcoding study of red fox and pine marten scats, we document a high occurrence of domestic dog DNA in red fox scats and investigate if it can be attributed to interspecific coprophagia. We tested whether experimental artifacts or other sources of DNA could account for dog DNA, regressed dog occurrence in the diet of fox against that of the fox’s main prey, short‐tailed field voles, and consider whether predation or scavenging could explain the presence of dog DNA. Additionally, we determined the calorific value of dog faeces through calorimetric explosion. The high occurrence of dog DNA in the diet of fox, the timing of its increase, and the negative relationship between dog and the fox's main prey, point to dog faeces as the source of DNA in fox scats. Dog faeces being highly calorific, we found that foxes, but not pine martens, regularly exploit them, seemingly as an alternative resource to fluctuating prey. Scattered accounts from the literature may suggest that interspecific coprophagia is a potentially frequent and widespread form of interaction among vertebrates. However, further work should address its prevalence in other systems and the implications for ecological communities. Tools such as metabarcoding offer a way forward.

## Graphical abstract

There is growing interest on the role facilitative interactions, but they remain underrepresented in the scientific literature and focused on plant communities. In a large‐scale metabarcoding study we report for the first time the frequent exploitation of domestic dog faeces by red foxes and argue that interspecific coprophagia is best described as an overlooked form of commensalism. These findings highlight how outdoor activities involving dogs may subsidize red fox populations, but also how interspecific coprophagia may have been overlooked as form of interaction among vertebrate animals.

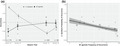

## 1 INTRODUCTION

Understanding how species interactions shape the composition and dynamics of communities is a central goal of ecology (Begon et al., 2005). Predators, parasites, parasitoids, and herbivores exert variable degrees of harm on their victims, and the consequences of such antagonistic interactions are generally well understood (Krebs, 2008). Other interspecific interactions that are not harmful for the individuals involved may amount to facilitation as either mutualism or commensalism (Mikula et al., 2018). Despite growing interest and the recognition of potentially far‐reaching implications for ecological communities, facilitation has received far less attention and has focused largely on plant communities (Bronstein, 2009; Bruno et al., 2003).

Among vertebrate animals, the provisioning of partly consumed carcasses to scavengers by larger predators is one instance of facilitation with implications for species interactions and coexistence (Moleón et al., 2014; Wilson & Wolkovich, 2011). However, determining the overall facilitative or antagonistic nature of carcass provisioning is not straightforward due to its dual nature with positive (food) and negative (mortality) impact on carcass users (Prugh & Sivy, 2020). Interspecific coprophagia, the ingestion of another species' faecal matter, could amount to a facilitative interaction among animals if faeces are an energetically and nutritionally valuable resource, are exploited as such, and the benefits of doing so outweigh the risks. However, the ecological role of faeces as a means of interaction has received little attention in vertebrate animals (but see Rowland, 1975).

Intraspecific coprophagia such as caecotrophy (i.e., consumption of one's own faeces fermented in the caecum) is well documented (see Hirakawa, 2001). In contrast, accounts of interspecific coprophagia remain scarce and often anecdotal, not least because of the empirical challenges of documenting it (e.g., Gallant, 2004; Nishikawa & Mochida, 2010; Yong, 2018). Like caecotrophy, interspecific coprophagia may fulfill species' essential needs. For instance, Egyptian vultures (*Neophron percnopterus* Savigny) are reported to obtain the carotenes necessary for their facial markings from ungulate dung pads (Negro et al., 2002). Similarly, small passerine birds have been hypothesized to obtain calcium from bones present in carnivore faeces (Gallant, 2004; Payne, 1972). Alternatively, species may engage in facultative coprophagia and consume faeces as they become available. Seabirds such as pale‐faced sheathbills (*Chionis albus* Gmelin), Wilson's storm petrels (*Oceanites oceanicus* Kuhl), kelp gulls (*Larus dominicanus* Lichtenstein), and dolphin gulls (*Leucophaeus scoresbii* Traill) have all been observed to feed on seal and whale faeces (Favero, 1996; Kraus & Stone, 1995; Seguel et al., 2017). Free‐ranging domestic dogs (*Canis lupus familiaris*) have also been documented to eat human faeces in India, Ethiopia, and Zimbabwe (Atickem et al., 2010; Butler et al., 2018; Vanak & Gompper, 2009). Crucially, human faeces were of similar or higher energy and nutritional value compared to other food items accessible to dogs (Butler et al., 2018). Furthermore, heterospecific faecal matter may provide alternative food sources when others are relatively unavailable. Reindeer in Svalbard (*Rangifer tarandus platyrhynchus* Vrolik) readily feed on barnacle goose droppings during the short summer season of the arctic archipelago, grotto salamanders (*Eurycea spelaea* Stejneger) in naturally oligotrophic caves switched to gray bat (*Myotis grisescens* Howell) guano during the latter's breeding season, and plateau pikas (*Ochotona curzoniae* Hodgson) extensively feed on domestic yak (*Bos grunniens* Linnaeus) faeces to survive the winter (Fenolio et al., 2005; Speakman et al., 2021; van der Wal & Loonen, 1998). Coprophagia in these disparate case studies takes place in conditions of local (Svalbard, caves) or seasonal (winter) food scarcity where faeces provide high‐value and digestible resources. Beyond scattered case studies, it is unknown whether interspecific coprophagia is an underappreciated yet ecologically relevant and widespread interaction within animal communities across biomes.

Quantifying the prevalence of interspecific coprophagia through direct observation or analysis of digestive tract contents and hard remains in faeces is rarely feasible, except where individuals form stable colonies, faecal matter remains identifiable (e.g., early digestion phase), or scats contain hard remains attributable to coprophagia (Butler et al., 2018; e.g., toilet paper, Nishikawa & Mochida, 2010; Seguel et al., 2017; Speakman et al., 2021). Instead, novel techniques relying on the presence of DNA in faecal samples (hereafter scats), such as metabarcoding, have the potential to infer coprophagia even in the absence of discernible remains (Speakman et al., 2021; Taberlet, Coissac, et al., 2012). This, however, remains largely untested.

In this study, we use a large metabarcoding analysis of the diet of mammalian carnivores in Scotland to explore whether dog faeces are an alternative food source for red fox (hereafter fox; *Vulpes vulpes* L.) when wild prey are scarce. We test several hypotheses that could explain the presence of dog DNA in fox scats: (i) consumption of dog faeces, (ii) dogs rolling or urinating over fox scats, or (iii) experimental artifacts of metabarcoding. Additionally, we discuss the implausibility of scavenging or predation of dogs as an alternative explanation in the context of the literature given the high occurrences of dog DNA observed in this study. Furthermore, we determine the calorific content of dog faeces through calorimetric explosion. Hypotheses (i) and (ii) are assessed by contrasting the probability of occurrence of dog and short‐tailed field vole (field voles hereafter; *Microtus agrestis* Linnaeus) DNA in fox scats over four sampling seasons and 2 years, and by regressing the former against the frequency of occurrence of the latter. A constant or seasonal occurrence of dog in the diet of fox with no relationship to the frequency of field vole in the fox's diet would support dogs tampering with fox scats as the source of dog DNA. Instead, an increasing or decreasing trend in dog occurrence in the diet of fox in response to changes in the field vole's occurrence would support consumption of faeces. For hypothesis (iii), we estimated the number of discrepancies between dog and fox DNA sequences, and visually explored the distribution of dog and field vole DNA sequence reads over that of fox. If dog detections are caused by consumption of faeces, the strength of the signal of dog (number of reads) should be unrelated to the signal of fox and the number of differences between the nucleotide sequences should be high.

## 2 METHODS

### 2.1 Study area

The study was based in the northwest of the Cairngorms National Park, Scotland (57°09′34″N, 03°51′40″W). The study area, with a temperate oceanic climate, is covered by seminatural (Caledonian) forest and a mix of Scot's pine (*Pinus sylvestris* L.) plantations and clear‐felled areas (for details see Zalewska et al., 2021). Field voles are the predominant prey species for many carnivores in the area, including red foxes (this study), and undergo cyclic changes in abundance (Lambin et al., 2000). Small mammal surveys indicated low field vole densities for the duration of the diet study (2018 and 2019).

### 2.2 Sampling

To study the diet of mammalian carnivores, scats were collected along transects on unpaved forest roads and trails in three to six sites (see below). Sites were 6–11 km^2^ and selected to maximize availability of transects and variability of habitats as determined by the proportion of clear‐felled and other herbaceous vs forest habitats, and deer‐culling pressure, which introduces carrion in the system. The minimum distance among neighboring sites' centroids ranged between 5 and 8 km. Sampling was done from February to April and from May to July in 2018, and again the same periods in 2019. These reflect ecologically relevant periods of food scarcity and the breeding season of prey of interest, to which we refer as winter and spring, respectively. Three sites were visited during the first season, six during the second season, and five during the following two seasons. The first season consisted of two visits, with samples collected in both, while the following seasons had three visits each where scats were cleared during the first one and collected in the following two to maximize collection of fresh samples. On average, visits were 21 days apart, ranging from 12 to 48 days. Tracks and trails were scanned by one or two surveyors walking abreast. Scats were identified to probable species in the field by size and shape (Summers et al., 2015). Approximately 1 cm^3^ of faecal matter was collected from 2887 samples using disposable wooden sticks and stored into 95% ethanol and then transferred to self‐indicating silica or directly stored into silica. Additionally, 298 samples were collected by volunteers. These were frozen whole and 1 cm^3^ transferred to silica up to 18 months later. A further 232 samples were collected by a preexisting and ongoing study in one of our study sites. These samples were also frozen whole and collected in a single visit coinciding with the last visit of our sampling seasons. From 3417 available samples, 2084 were selected for metabarcoding analysis to maximize spatial and temporal coverage of independent meals. The selected samples included all samples putatively identified in the field as fox (763) or Eurasian badger (*Meles meles* L.; 85), and a subset of the samples tentatively identified in the field as pine marten (*Martes martes* L.; 973), least weasel or stoat (*Mustela erminea* and *Mustela* nivalis L.; 213), and unidentified samples (50). For additional details on the selection of samples, see Text S1 in Appendix S1.

### 2.3 DNA metabarcoding

We analyzed the vertebrate component of the diet of mammalian carnivores through DNA metabarcoding (Shehzad et al., 2012). Total DNA was extracted to a final volume of 400 μl using the NucleoSpin Soil Kit (Macherey‐Nagel, Germany) after mixing samples with a sodium phosphate lysis buffer for 15 min (Taberlet, Prud'Homme, et al., 2012). The 12S mitochondrial rRNA gene was then amplified by triplicate using the universal vertebrate primer 12SV5 (Riaz et al., 2011). Amplification of fox, marten, and badger DNA was only partly blocked through blocking oligonucleotides to allow host identification (See Text S2 in Appendix S1; Vestheim & Jarman, 2008). Extraction and amplification were carried out in dedicated and separate rooms. Sequencing was outsourced to Fasteris (Geneva) and done using a NextSeq 500 (Illumina, USA).

Sequencing files were analyzed using OBITools (Boyer et al., 2016). Sequences found only once in the dataset, containing degenerated bases or that were too short or long (<60 bp, >130 bp) were removed first. Molecular taxonomic units (MOTUs) that did not reach 10 reads in at least one PCR were removed too. The remaining were then taxonomically annotated by comparing against a local and a global reference database prepared from the EMBL's European Nucleotide Archive (http://www.ebi.ac.uk/embl/). Only alignments with at least 95% sequence identity were kept in the final data. Contamination (mostly primate DNA) and tag jumps (i.e., sequences assigned to the wrong PCR during sequencing) were identified at this stage and removed. Samples were assigned to carnivore hosts through a “voting system” wherein a carnivore had to be the most common of all potential hosts in at least two of the three PCR replicates while also representing at least 1% of the PCR's reads. The resulting data were manually curated to address imperfect alignments, assignations to non‐native or redundant taxa (e.g., *Microtus* and *Microtus agrestis*), or not at species‐level. Where multiple MOTUs were assigned to the same taxon and present in the same PCR their reads were combined. Only detections that represented at least 1% average relative frequency of reads across amplifications replicates were used in later analysis (Deagle et al., 2019). For additional details on the laboratory, bioinformatic and manual curation process, see Appendix S1 (Text S2).

### 2.4 Analysis

Both percentage frequency of occurrence (proportion of positive scat samples; % FO) and a modified relative read abundance (RRA), average percentage of reads of a given taxon across *positive* scat samples, were used to summarize dog and field vole occurrence in the diet of fox and marten.

To test whether foxes exploited dog faeces as an alternative food source to voles, we modeled the probability of occurrence of dog and field vole in the diet of fox as binomial random variables over time (the four sequential seasons fitted as a categorical variable), and regressed dog probability of occurrence in the diet of fox over field vole % FO, in three generalized linear models. Sampling season and field vole % FO covaraites were fitted in separate models due to strong colinearity. Within each season, variables were aggregated at site level (three to six site levels per season). Sites were considered independent replicates after testing for spatial autocorrelation at sample and site level using the “*testSpatialAutocorrelation*” function of the “*DHARMa*” R package (Florian, 2021; R Core Team, 2021). Two models were used for this, one with a binary binomial variable (presence‐absence of dog DNA) and sampling season as a categorical covariate and another one with the same variables but a random intercept of site (fitted with “*glmer*” from the package “*lme4*”; Bates et al., 2022). Uniformity and overdispersion of the residuals and presence of outliers were tested using “*DHARMa*.” Quantile deviations were observed for the field vole model but were overall not significant and model assumptions were met. Model estimates are presented alongside their 95% confidence interval.

To address whether dog DNA sequences were artifacts of fox sequences, differences between the sequences of MOTUs assigned to fox (*n* = 6) and dog (*n* = 21) were calculated using the “*adist()*” of the “*utils*” package. The number of dog and field vole DNA sequence reads per PCR (*n* = 1941; 647 fox scats x 3 amplification replicates) were plotted against the number of fox reads per PCR and fitted with 90th quantile regressions over the nonzero component using the “*rq*” function of the “*quantreg*” R package (Koenker, 2021). The number of sequence reads (plus the smallest read count found in the data) were log transformed (base 10) before plotting and fitting the quantile regression. Data management, analysis, and visualization was made with R 4.0.5.

### 2.5 Calorimetry

To obtain a measure of the energetic content of fresh dog faeces, samples from six dogs and households that consumed a range of dry and wet, commercially available, dog food were analyzed through calorimetric explosion (Hambly & Speakman, 2005). Samples were weighted before and after drying at 60°C for 14 days to calculate the percentage water content in the samples. They were then homogenized in a blender and compressed into pellets of 0.15–0.25 g and exploded in a Parr 6100 calorimeter using a 1108 oxygen bomb (Parr Instrument Company, USA) after calibration using benzoic acid. Each sample was replicated 2–4 times until the relative standard deviation was <1.5% of the mean value. Results are presented per MJ kg^−1^ of dry and wet weight and as kilocalories per 100 g of dry and wet weight. Noncombustible mineral residual material was weighed for four of the six dogs and presented as the % weight of the wet and dry pellets. Wet weight of the pellets was back transformed from the dry weight using average water content of this study's dog faeces (60.5%).

## 3 RESULTS

A total of 647 samples were genetically identified as red fox and yielded diet data. Domestic dog was detected in 39.1% samples while field vole occurred in 55.2% (Table 1). The RRA was 26.5% for dog and 65.4% for field vole. In the diet of pine marten (*n* = 1060), dog was detected at 0.85% FO and field vole at 56.51% FO. RRA's were 14.6% and 71.21%, respectively.

**TABLE 1:** Frequency of occurrence (% FO) and modified relative read abundance (RRA) of field vole and domestic dog in the diet of red fox and pine marten

Predator	Prey	No of occurrences	*N*	FO (%)	RRA (%)
*Vulpes vulpes*	*Microtus agrestis*	357	647	55.18	65.38
	*Canis lupus familiaris*	253		39.1	26.51
*Martes martes*	*Microtus agrestis*	599	1060	56.51	71.21
	*Canis lupus familiaris*	9		0.85	14.6

### 3.1 Are dog DNA sequences an artifact?

**FIGURE 1:**
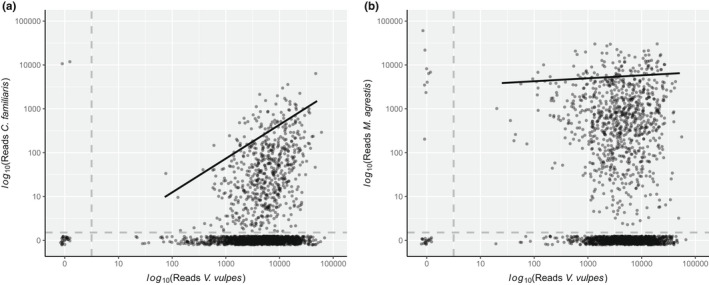
Number of DNA sequence reads of domestic dog (a) and field vole (b) over DNA sequence reads of red fox at PCR level (*n* = 1941; 647 scats × 3 amplification replicates). Data were transformed with a base 10 logarithm after adding one read to all samples. Gray dashed lines divide the data into four areas: No reads of either species (bottom left), positive for dog (a) or vole (b) reads but no fox reads (top left), negative for dog/vole reads but positive for fox (bottom right) and positive for reads of either species (top right). Solid black lines represent the 90th quantile regressions over non‐zero data

The number of differences between fox and dog sequences averaged 7.68 (± 1.73) nucleotides and ranged from 4 to 12. The maximum number of dog sequence reads per PCR (90th quantile) increased with increasing fox reads (Figure 1a). This is caused by a relationship between the maximum number of reads one taxon (including the host) can reach in a PCR and the total number of reads of the PCR, whereby the former increases with the latter (Figure S1 in Appendix S1). Additionally, 66% of the PCRs had no dog reads and were distributed throughout the range of fox reads (Figure 1a). This pattern was akin to the fox's main prey, field voles, also absent in 52% of the PCRs (Figure 1b).

### 3.2 Are dog faeces used as an alternative resource?

**FIGURE 2:**
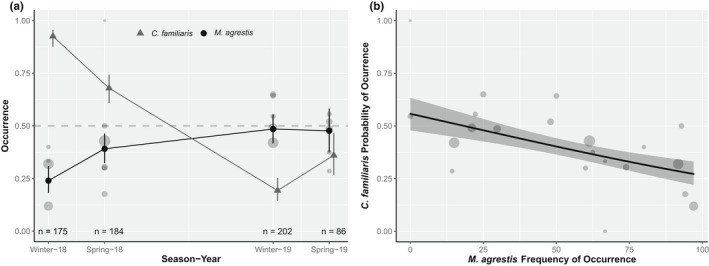
Generalized liner model outputs. (a) Predicted probability of occurrence of domestic dog DNA (black circles) and field vole (gray triangles) in scat samples of red fox at each sampling season and across sites. Line ranges represents 95% confidence interval. The number of fox samples in each season is printed at the bottom of the panel. (b) Predicted probability of occurrence of domestic dog in red fox samples over field vole frequency of occurrence. Shaded area shows 95% confidence interval. Raw data of dog frequency of occurrence plotted as gray circles weighted by sample size in both panels

The average probability of occurrence of dog DNA in fox scats in winter 2018 was 0.24 (0.18–0.31), significantly lower (*p* < .01; Table S1 in Appendix S1) than the following three seasons, at 0.39 (0.32–0.46), 0.49 (0.42–0.55) and 0.48 (0.37–0.58) in spring 2018 and winter and spring 2019, respectively (Figure 2a). The average probability of occurrence of field vole DNA in fox scats in winter 2018 was 0.93 (0.88–0.96), significantly higher (*p* < .01; Table S1 in Appendix S1) than the following three seasons, being at 0.68 (0.61–0.74) in spring 2018, reaching its lowest in winter 2019 (0.19, 0.14–0.25), and slightly rebounding to 0.36 (0.27–0.47) by spring 2019 (Figure 2a). The probability of dog occurrence in the diet of fox declined significantly with increasing field vole frequency of occurrence (*p* < .01; Table S1 in Appendix S1), from 0.56 (0.48–0.63) in the absence of vole down to 0.27 (0.22–0.33) at 97% vole FO (Figure 2b).

### 3.3 Are dog faeces energetically valuable?

On average, dog faeces contained 135.02 kcal per 100 g of wet weight (345.57 kcal per 100 g of dry weight; Table 2). Additionally, 12.1% (7.6%–19.5%) of the wet weight of pellets from four of the six dogs analyzed consisted of noncombustible mineral materials (30.7% of dry weight; 17.7%–51.8%).

**TABLE 2:** Calorific content of domestic dog faeces estimated from samples of six dogs from six households (*N*)

Species	*N*	Megajoules per dry kg	Kilocalories per dry 100 g	Megajoules per wet kg	Kilocalories per wet 100 g
*Canis lupus familiaris*	6	14.47 ± 2.20	345.57 ± 52.52	5.65 ± 1.65	135.02 ± 39.34

	*N*	Proportion of mineral content	
		% weight of dry pellet	% weight of wet pellet
*Canis lupus familiaris*	4	30.7	12.1

*Note:* Figures as presented as megajoules per kg of dry and wet weight and as kilocalories per 100 g of dry and wet weight. Proportion of mineral content of pellets estimated as the average percentage weight of non‐combusted material out of the dry and wet weight of the pellet. Wet pellet weight was back transformed from dry weight using average water content of this study's dog faeces (60.5%). Mineral data available only from four dogs.

## 4 DISCUSSION

Our data are consistent with domestic dog faeces being a valuable alternative food source for wild red foxes, with potentially important implications. Domestic dog was the second most frequently detected taxon in the diet of foxes, being globally present in 39% fox scats and raising to 48.5% during the season with the lowest occurrence of field voles. Although promoting recreational activities such as dog walking is one of several management targets in the study area, which also include timber production and wildlife conservation, dog walking may provide a previously unrecognized alternative exogenous resource to red foxes. We propose that in this context, facultative interspecific coprophagia is best described as a form of commensalism. If this behaviour extends beyond human–animal interactions, it could be an underappreciated form of commensalism. Moreover, if widespread, such an interaction among animals could complexify species interaction networks, connecting otherwise independent species, and operate as a stabilizing force against environmental fluctuations. However, grasping the magnitude of these implications is contingent on a better understanding of its prevalence across ecological communities.

The occurrence of dog DNA in fox scats was not the result of metabarcoding artifacts. The differences between dog and fox sequences (>4%) were beyond the error rates expected from our sequencing runs. Averaging across the four sequencing lanes, 88% of DNA reads had a 0.1% or less error rate. Additionally, the occurrence and number of dog DNA sequence reads was unrelated to fox. Both results point to genuine dog DNA as the source. Other sources of dog DNA such as dog urination or rolling over fox scats cannot be formally discounted. However, given the timing of the increase in occurrence of dog DNA and the negative and significant relationship with field vole occurrence, the hypothesis of coprophagia is better supported. Additionally, these results suggest the exploitation of this exogenous resource is driven by the low availability of the main prey.

Although technically not impossible, we did not consider the possibility of predation or scavenging of domestic dogs as a sufficient explanation. Dog has been documented in the diet of red foxes in their invasive range, Australia, with indirect evidence pointing to domestic dogs rather than the closely related dingo (Brunner et al., 1991; Fleming et al., 2021). However, where dog is reported, it is generally attributed to scavenging of roadkill, albeit coprophagia is not ruled out. Moreover, the frequencies of occurrence reported are smaller than in the present study (e.g., 0.34% FO; Fleming et al., 2021). Therefore, we posit that the high frequencies observed here (39.1% FO) would require either an implausibly high availably of dog carrion (e.g., roadkill left unattended), frequent instances of dog predation, or both, which would not go unnoticed by the public and media (see Murugesu, 2021). Furthermore, the low proportion that dog DNA reads present in samples positive for dog relative to the same figures for field vole suggests a DNA‐poor source and is congruent with the ingestion of faeces rather than DNA‐rich tissue (Table 1). Collectively, although some uncertainty on its true rate remains, we confidently conclude that our analyses reveal coprophagia of domestic dog faeces by red foxes.

Metabarcoding has enabled the detection of an otherwise overlooked food source in the diet of the red fox, an intensely studied species that often coexists closely with humans and their dogs (Handler et al., 2019; Soe et al., 2017). The lack of hard remains of the average domestic dog stool and the generalist diet of foxes overlapping with many sympatric species renders the detection of interspecific coprophagia through mechanical sorting of prey remains virtually impossible (Macdonald, 1987a; Tercel et al., 2021). Nonetheless, three previous metabarcoding studies of red fox diet have not reported occurrence of dog either (Nørgaard et al., 2021; Shao et al., 2021; Xinning et al., 2019). This might be related to the prevalence of dog walking in their study areas or to smaller sample sizes (*n* = 72, 3 and 103 scats, respectively, vs. 647 here). Alternatively, domestic dog, like human DNA in this study, may have been dismissed as contamination a priori. Interestingly, human faeces are also available throughout our study area (e.g., wild campers) and could have contributed to human DNA presence in the samples. Where direct predation can be discarded, metabarcoding offers the means to identify otherwise cryptic interspecific coprophagia, and the use of bioinformatic filters should be conscious of this possibility. Nonetheless, further work should consider empirical validation by pairing it with direct observation or experimental designs (e.g., contrasting with dog‐free areas; van der Wal & Loonen, 1998).

That interspecific coprophagia has gone unnoticed in such an intensely studied species opens the possibility of it being geographically and taxonomically widespread. For instance, in addition to the literature showcased in the introduction, nine species of vertebrates were reported feeding at giant otter (*Pteronura brasiliensis* Gmelin) latrines in Brazil (Leuchtenberger et al., 2012). One could envisage a scenario where most species facultatively exploit the faeces of sympatric species. Certainly, we also detected pine marten (0.62% FO) and Eurasian badger (2.16% FO) DNA in the diet of foxes, dog (0.85% FO) and fox (0.57% FO) DNA in the diet of marten, and marten DNA (2.44% FO) in the diet of badger (*n* = 41 scats). While badger DNA in the diet of fox followed a similar temporal pattern to that of dog (ranging between 1.14 and 4.65% FO), the overall low occurrences and inability to discard events of predation or scavenging, prevent further inference (Kidawa & Kowalczyk, 2011). That a wild canid, but not two mustelids, regularly exploited faeces of a domestic canid despite experiencing the same availability of faeces and fluctuating vole prey may suggest coprophagia is more likely between taxonomically related species. However, with documented case studies including ungulates, birds, mustelids or primates, the literature does not support this hypothesis (Gallant, 2004; Negro et al., 2002; Nishikawa & Mochida, 2010; van der Wal & Loonen, 1998). Instead, coprophagia seemingly occurs between animals with overlapping trophic niches, with herbivores more likely to exploit the faeces of other herbivores (e.g., Speakman et al., 2021), or piscivores exploit other piscivores (e.g., Favero, 1996). An additional, but nonexclusive, scenario contrasts coprophagia among wild species to the exploitation of exogenous resources introduced by humans (including faeces), which few synanthropic species may monopolize (Atickem et al., 2010; Vanak & Gompper, 2009).

Dog faeces had a considerable calorific content. Per 100 g of live weight (135.02 kcal), it was comparable to that of a range of small rodents (137–170 kcal per 100 g of live weight; Fleharty et al., 1973). Considering an average Scottish adult fox of 6.4 kg, their daily energy needs would oscillate between 400 and 800 kcal per day depending on the season (Kolb, 1978; Saunders et al., 1993). This would require from 10 to 30 small rodents per day (assuming an average weight of 20 g per rodent), or 300 to 600 g of dog faeces per day to satisfy the needs of an average adult fox. Given the virtually null foraging costs of dog faeces compared to wild prey, the potential energy gain from interspecific coprophagia is substantial. Additionally, owing to their nutrient rich diets, dog faeces may also have high amino acid, vitamin, and mineral contents (Table 2; Davies et al., 2017; Flachowsky, 1997). Furthermore, faecal matter may be more digestible than some raw foods (Krief et al., 2004; Speakman et al., 2021). Although we cannot estimate the size of the input of faecal matter into the system, the number of visitors to the Cairngorms National Park has increased from 1.3 million in 2004 to 2.1 million in 2019 (MacDonald, 2020). Combined with high occurrence in the diet of foxes, it suggests dog faeces may be a rich, (increasingly) abundant, and inert, resource.

Red foxes generally specialize in small rodents when these are abundant and switch to alternative prey when they are scarce (Breisjøberget et al., 2018; Kjellander & Nordström, 2003). In productive areas, prey switching may allow them to decouple from the demographic cycles of their main prey (Breisjøberget et al., 2018; Erlinge, 1987). Certainly, foxes thrive in human‐modified landscapes, where they are reported to have small home ranges, and exploitation of human subsidies is part of their adaptive strategy (Handler et al., 2019; Jahren et al., 2020; Macdonald, 1987b). Increasing recreational activities in remote areas may inadvertently subsidize fox populations and contribute to decouple them from their main prey even in low productivity areas. The potentially detrimental effects of such subsidies on the fox's competitors and prey should be considered alongside direct disturbances to wildlife (Hughes & Macdonald, 2013; Shutt & Lees, 2021).

The ingestion of faeces poses an obvious risk of pathogenic or parasitic infection due to potentially high infectious loads in animal faeces, including dog's faeces (Otranto et al., 2015; Raue et al., 2017; Sager et al., 2006; Xhaxhiu et al., 2010). Over time, the accumulated risk of exposure to at least one parasitic helminth or protozoan (or other pathogens such as canine distemper) through interspecific coprophagia is likely high, with high incidences of some of these pathogens reported in fox populations in Europe and the United Kingdom (Gillespie et al., 1956; Otranto et al., 2015; Richards et al., 1995; Sager et al., 2006). In a context of human and animal global travel, coprophagic behaviors may expose wild animals to new pathogens (Messenger et al., 2014). However, such risks should be assessed against the risks of not consuming faeces, which may include starvation, and the threat of infection from other pathways. For instance, like other disease agents, parasites such as *Echinococcus multilocularis* can be transmitted through the consumption of intermediate or paratenic hosts (in addition to direct faecal‐oral pathways), and others yet are transmitted by arthropod vectors (Otranto et al., 2015; Raoul et al., 2015). Additionally, wild animals can often tolerate moderate parasite loads without manifesting symptoms (Thompson et al., 2010).Thus, interspecific coprophagia may represent a relatively small added cost when set against real energy gains (see above). Indeed, the studies of coprophagia that assessed the health of the individuals involved found no associated pathologies, although the evidence remains scarce (Butler et al., 2018; Krief et al., 2004). Conversely, individuals may be disturbed or injured by the coprophage. When documented, these are rather benign and unlikely to bear on the population (Nishikawa & Mochida, 2010; Seguel et al., 2017; van der Wal & Loonen, 1998). Therefore, we postulate that in this instance, the potential gains of interspecific coprophagia appear to outweigh its potential drawbacks and propose that facultative interspecific coprophagia is best described as a form of commensalism. However, the balance between benefits and risks will vary according to the diversity and incidence of pathogens circulating and, where domestic dogs are involved, to the vaccination status of the dog population.

## 5 CONCLUDING REMARKS

In this study, we report an overlooked pathway of interaction between wildlife and human activities, wherein red foxes in Scotland extensively consume highly calorific dog faeces as an alternative food to fluctuating wild prey. The deposition of large amounts of faecal matter and other waste (e.g., urine) is likely to have extensive effects beyond vertebrate wildlife, including invertebrates, plants and soil microorganisms, which should be considered when planning and implementing management interventions (de Frenne et al., 2022). The evidence presented here, and other accounts scattered in the scientific literature, hint at the possibility that interspecific coprophagia is also an underappreciated but widespread form of commensalism among vertebrate animals, whereby some species may enable others to access resources unavailable to them, potentially buffering them against temporal fluctuation in resource availability (e.g., ungulates consuming droppings from macaques that fed in the tree canopy; Nishikawa & Mochida, 2010). However, we anticipate that even if common, interspecific coprophagia would be loosely restricted to members of the same trophic guild (e.g., piscivores, herbivores). Should this be confirmed, such an interaction could shift our understanding of the properties of vertebrate species assemblages such as connectance, modularity, robustness, or stability. (Bruno et al., 2003; Grilli et al., 2016; Jacquet et al., 2016; McCann et al., 1998; Pocock et al., 2012; Stachowicz, 2001). More data of the kind provided here are required to evaluate the hypothesis of widespread coprophagia and better understand its wider ecosystem implications. Metabarcoding can contribute to the study of such an elusive interaction.

## AUTHOR CONTRIBUTIONS

**Cristian D. Navarro Waggershauser:** Conceptualization (equal); data curation (lead); formal analysis (lead); investigation (lead); methodology (equal); resources (equal); software (equal); writing – original draft (lead); writing – review and editing (equal). **Pierre Taberlet:** Investigation (equal); methodology (equal); resources (equal); supervision (supporting); writing – review and editing (supporting). **Eric Coissac:** Data curation (equal); investigation (equal); methodology (equal); software (equal). **Kenny Kortland:** Funding acquisition (equal); project administration (equal); resources (equal); writing – review and editing (equal). **Catherine Hambly:** Investigation (equal); methodology (equal); resources (equal); supervision (supporting); writing – review and editing (supporting). **Xavier Lambin:** Conceptualization (supporting); funding acquisition (equal); investigation (supporting); methodology (supporting); project administration (equal); resources (equal); supervision (lead); writing – review and editing (equal).

## CONFLICT OF INTEREST

There are no coflicts of interest to declare.

## Supporting information


Appendix S1

## Data Availability

The data and code used in this study is presented within or available from the Dryad Data Repository: https://doi.org/10.5061/dryad.qz612jmj4.
