# Supplementary material for: Interspecific coprophagia by wild red foxes: DNA metabarcoding reveals a potentially widespread form of commensalism among animals
Source: Ecol Evol. 2022 Jul 3;12(7):e9029. doi: 10.1002/ece3.9029 (PMC9251403; doi:10.1002/ece3.9029)
Supplement: Supplementary file 1 — Appendix S1 [file ECE3-12-e9029-s001.docx]

**APENDIX 1**

**Supplementary Text S1: Methodological details of sample selection**

Samples were selected according to the priorities of the wider diet analyses of which we present a small component in this study. To maximise detectability of instances of intraguild predation and rare predation events of prey of conservation concern, all putative samples of red fox were selected for further analysis (n = 737). Eurasian badger samples too were all selected for further analysis to address the presence of ground nesting birds in their diets (n = 85). A further 70 samples (26 fox, 31 marten and 13 putative weasel or stoat) from a neighbouring estate were analysed here too. Instead, owing to their abundance, pine marten samples were subsampled from the available pool. This was achieved with two separate methods that selected samples from segments of the transects (400 to 800 meters; McHenry et al., 2011). The first prioritised spatial and temporal coverage by selecting one sample from each segment within a site, visit, season and year combination, yielding 731 sample. Instead, the second method prioritised spring and fresh and intact samples by randomly selecting them out of the remaining pool, regardless of which transect segment they belonged to, yielding 211 samples. Another 200 putative samples of weasel or stoat samples, and 50 unidentified samples were also put forward for metabarcoding analysis. The former were included to roughly characterise the diet of small mustelids in the area, and the latter to fully parameterise field identification success rates. Both were allocated in rough proportion to their occurrence in the pool of samples. Selection of weasel or stoat samples prioritised spatial coverage while selection of unidentified samples prioritised sample quality. The method described here *de facto* maximised sampling effort on site-visit combinations with fewer samples.

**Supplementary Text S2: Methodological details of metabarcoding**

**DNA Extraction**

Prior to extraction, approximately 1 cm^3^ of sample was grinded with a clean surface and introduced in a falcon tube. Between 5 and 15 mL (depending on volume and composition of sample) of phosphate lysis buffer was added to the sample, consisting of 1 L of water (Evian 1 L bottle) saturated with 0.10 M Na_2_HPO_4_ and 0.016 M NaH_2_PO_4_ for a final pH of ca. 8. Mixing lasted 15 minutes and was done with an Intelli-Mixer (ELMI, Latvia). Total DNA was then extracted using the NucleoSpin Soil Kit (Macherey-Nagel, Germany) following manufacturer instructions except for modified volumes for the binding buffer (SD: 265 µl) and the last two washing buffers (SW2: 750 µl). Extracts were recovered in a total volume of 400 µl. An extraction control with no sample was used every 47 samples (44 in total) to monitor contamination during extraction stage.

**DNA Amplification**

The 12S mitochondrial rRNA gene was amplified using the universal vertebrate primer 12SV5 (Riaz et al., 2011). A double four by three 96-plate system was used (two sets of 12 plates). Each row and column within a set of 12 plates combination had a unique forward and reverse primer tag combination (36 forward and 32 reverse tags; 7-8 bp long with at least 5 differences between them) to identify samples. Tags were randomized at plate level between the three PCR replicates. Each 12-plate set was later tagged with unique Illumina adapter during library preparation. Additionally, each forward and reverse tag was not used at least once in each row and column of the 12-plate design to control for tag-jumps. A total of 88 tag ‘blanks’ were used per PCR replicate (24 plates). Positive controls with a known source of DNA and PCR negative controls with clean water as DNA template were also used every 47 samples to monitor contamination during the amplification stage and aid bioinformatic filtering.

Amplifications were done in a final volume of 20 µl comprised of 10 µl of AmpliTaq Gold 360 Master Mix (Applied Biosystems, USA), 0.16 µl of bovine serum albumin, 0.8 µl of primers (final concentration 0.2 µM), 0.4 µl of blocking oligonucleotides (final concentration 2 µM, equally divided between the three blockers: fox, marten and badger) and 6.64 µl of water. The extract was diluted four times and 2 µl of template DNA were added to the mix. Amplification started with 10 minutes at 95 °C, followed by 45 cycles of 30 seconds at 95 °C, 30 seconds at 49 °C and 1 minute at 72 °C. Six plates containing samples assigned as ‘old’ in the field were left for 50 cycles to maximise DNA amplification. The presence of DNA in the PCR product was asserted via electrophoresis gel, purified using QGIAxcel (QIAgen, USA) and quantified with Qubit (Thermo Fisher Scientific, USA) before being sent to Fasteris (Geneva) for sequencing with a NextSeq 500 (Illumina Inc., USA). Fasteris uses the MetaFast protocol for library preparation ([www.fasteris.com](http://www.fasteris.com)).

Amplifications and extractions were done in dedicated, separate rooms.

**Bioinformatic pipeline**

A total of 68,213,199 sequences were obtained. The sequencing files were analysed using OBITools (<https://git.metabarcoding.org/obitools>). Sequences were sorted through tag and primer sequences with *‘ngsfilter’*, allowing up to 2 errors in the primers. Sequences occurring only once in the dataset, with degenerated bases or too short or long (< 60 bp, > 130 bp) were removed. Molecular taxonomic units (MOTU’s) that did not reach 10 reads in at least one PCR were removed too. These filters removed 4,345,078 sequences. Another 2,379,111 potential PCR errors were then discarded using *‘obiclean’*, which clusters sequences that have no more than 1 bp difference, and retains only the most abundant sequence from each cluster. Taxonomic annotation was done through a global alignment algorithm against a reference database with *‘ecotag’* and a minimum identity of 85%. Two reference databases were prepared by extracting the region of interest of the 12S mitochondrial gene from the EMBL’s European Nucleotide Archive (<https://www.ebi.ac.uk/ena>). A local database with only vertebrate species present in the study area and a second global database with all recorded vertebrate. The local list of species was built from local expertise, atlases as well as all records from the National Biodiversity Network with more than 10 accounts (<https://nbnatlas.org/>). Only alignments with at least 95% identity were kept in the final dataset. Primate DNA and sequences assigned to an undetermined taxon (taxon:1) were considered contaminants and removed, 57,562,820 sequences remained. Tag jumps were also identified and filtered out, deleting another 2,329,881 sequences.

Samples were assigned to one of 10 possible hosts (*Anser, Canis, Erinaceus, Felis, Lutra, Martes, Meles, Mustela erminea, Mustela nivalis and Vulpes*). To be assigned, a potential host had to be the most abundant of the 10 in at least two of the three PCR replicates while representing at least 1% of the PCR’s reads. Hosts comprised 34,609,884 sequences, leaving 20,623,055 after their removal. Relative frequencies of prey are estimated after host deletion. Out of 2,084 samples analysed, amplification and sequencing were successful in 1,921 of which 1,776 could be assigned to a host: 1,060 pine marten, 647 red fox, 41 Eurasian badger, 15 domestic dog, three otter and two cat (wild or feral). Another eight samples (2 dog, 2 badger and 4 marten) were also assigned to host but contained no prey sequences.

**Manual curation**

Following bioinformatic filtering, the dataset was manually curated. A total of 324 MOTU’s were identified and taxonomically annotated as per above. However, these included imperfect assignations (<100% identity), assignations that were not at species level, redundant taxonomic assignations (i.e., different MOTU’s assigned to the same taxon) and assignations to non-native taxa. These were explored and where fitting, re-assigned. Where a MOTU was assigned to a non-native taxon by the global database, but to a suitable taxon (>95% identity) by the local database, the latter was kept (e.g., *Talpa occidentalis* to *Talpa europaea*). Where neither database offered a suitable alternative, the sequence was run on NCBI’s Nucleotide BLAST (<https://blast.ncbi.nlm.nih.gov>) and re-assigned to a suitable taxon. If BLAST did not offer a suitable alternative, the MOTU was re-assigned to a higher taxonomic level that contained a native species (e.g., *Eliomys melanurus* are absent in Britain, and their sequence reads were re-assigned to Rodentia). If a MOTU had been assigned to a non-species level taxon, but this one contained a single native species in the study area, it was automatically re-assigned (e.g., *Apodemus* to *Apodemus sylvaticus*). If said taxon comprised multiple native species, they were re-run using BLAST. Lastly, all species-level taxon assignations with imperfect alignments were checked again using BLAST (only one case of re-assignation at this stage). Before manual curation, species-level assignations comprised 47% of MOTU’s and 70.5% were assigned to genus level. After manual curation, these figures raised to 77.2% and 85.1%, respectively. MOTU’s assigned to the same taxon and present in the same sample were merged.

**Primers and Blocking Oligonucleotides**

| *primers* | |
| --- | --- |
| Forward | TTAGATACCCCACTATGC |
| Reverse | TAGAACAGGCTCCTCTAG |
| *blocking oligonucleotides* | |
| *Martes* | ctatgcCCAGCCCTAAACACAAACAATTTACAC-C3 |
| *Meles* | ctatgcTCAGCCCTAAACATAGATAATTCATAG-C3 |
| *Vulpes* | ctatgcTTAGCCCTAAACATAAATAGTTCTATA-C3 |

**References**

Riaz, T., Shehzad, W., Viari, A., Pompanon, F., Taberlet, P., & Coissac, E. (2011). ecoPrimers: inference of new DNA barcode markers from whole genome sequence analysis. *Nucleic Acids Research*, *39*(21), e145–e145. <https://doi.org/10.1093/NAR/GKR732>

McHenry, E., O’Reilly, C., Sheerin, E., Kortland, K., & Lambin, X. (2016). Strong inference from transect sign surveys: combining spatial autocorrelation and misclassification occupancy models to quantify the detectability of a recovering carnivore. *Wildlife Biology*, *22*(5), 209–216. <https://doi.org/https://doi.org/10.2981/wlb.00146>

**Supplementary Table S1**

Table S1. Coefficient table of generalised linear models contrasting Probability of Occurrence of domestic dog and field vole in red fox scat samples at each sampling season (Model 1 and 2, respectively; panel A in Figure 1) and regressing dog Probability of Occurrence over field vole Frequency of occurrence (Model 3; panel B in Figure 1). Degrees of freedom of each model are printed between parenthesis next to model title.

| *covariate* | | *estimate* | | *std.error* | *z.value* | | *p.value* | | |
| --- | --- | --- | --- | --- | --- | --- | --- | --- | --- |
| Model 1 (17 *d.f.*) | | | | | | | | | |
| Intercept | | -1.15 | | 0.18 | -6.51 |  | | |  |
| Spring-18 | | 0.71 | | 0.23 | 3.05 | < 0.01 | | |  |
| Winter-19 | | 1.09 | | 0.23 | 4.83 | < 0.01 | | |  |
| Spring-19 | | 1.06 | | 0.28 | 3.80 | < 0.01 | | |  |
| Model 2 (17 *d.f.*) | | | | | | | |  |  |
| Intercept | 2.52 | | 0.29 | | 8.75 |  | |  |  |
| Spring-18 | -1.77 | | 0.33 | | -5.39 | < 0.01 | |  |  |
| Winter-19 | -3.95 | | 0.34 | | -11.66 | < 0.01 | |  |  |
| Spring-19 | -3.10 | | 0.37 | | -8.47 | < 0.01 | |  |  |
| Model 3 (19 *d.f.*) | | | | | | | |  |  |
| Intercept | | 0.23 | | 0.16 | 1.44 |  | | |  |
| *M. agrestis* % FO | | -0.01 | | 0.00 | -4.75 | < 0.01 | | |  |

**Supplementary Figure S1**

Figure S1. Number of sequence reads per PCR of domestic dog (A), field vole (B) and red fox (C) over total number of reads per PCR. Data is transformed with a base 10 logarithm. Zeroes were added one read prior to transformation. Grey area highlights the 1:1 relationship area.


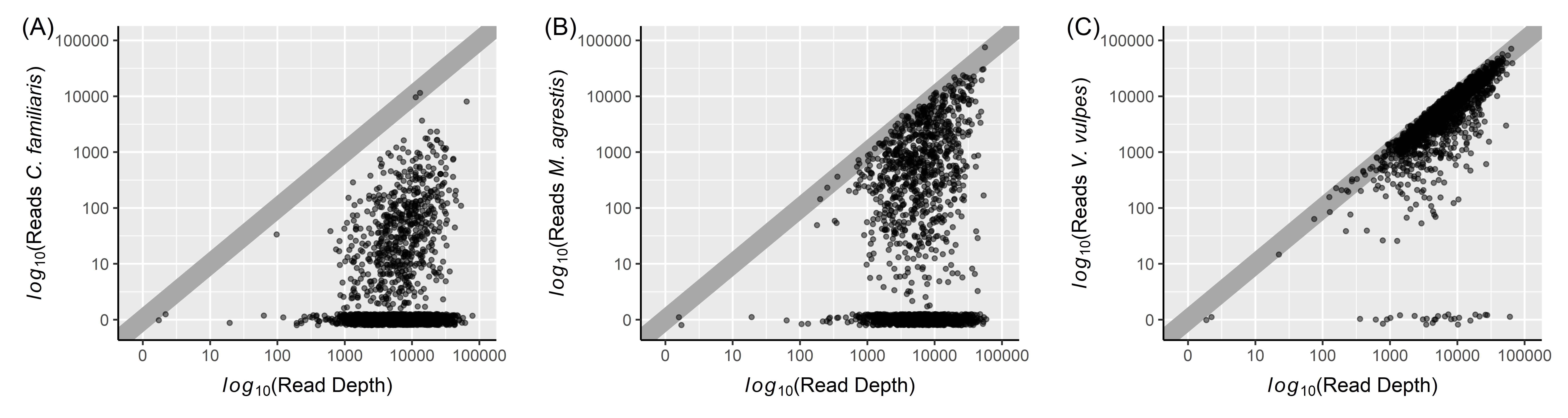


**APENDIX 2**

**Supplementary Text S1: Testing Domestic Dog Co-occurrence with Other Domestic Prey**

Following comments by PhD examiners, the following analysis is appended to this manuscript for transparency.

If red foxes are consuming large amounts of domestic dog faecal matter, food items commonly found in dog foods such as chicken, lamb or beef could be identified in the diet of foxes, in association with the presence of dog DNA. Certainly, these food items were identified: *Gallus* 3.6% FO; *Salmo* 0.8% FO; *Bos* 2.8% FO; *Sus* 2.6% FO; *Ovis* 3.3% FO. Their association with dog DNA was tested fitting a general linear model with a binary binomial variable depicting the presence of any of five categories of prey (*Gallus, Salmo, Bos, Sus, Ovis*) as 1, and their absence as 0, in fox scat samples. This was regressed against the presence or absence of domestic dog in the same samples. Model assumptions were validated with ‘*DHARMa*’. There was no significant effect of dog on the Probability of Occurrence of ‘domestic’ prey (Figure S1). The absence of an effect does not support interspecific coprophagia of dog faeces by red foxes. However, it is uncertain what DNA-load such prey would have in fox scats. Meat in processed dog foods is often cooked, which would be then digested by dogs, exposed to the environment, digested by foxes and exposed to the environment again until sampling. Furthermore, foxes may have independent access to these food items given fisheries, livestock, and different forms of waste can be present throughout the study area.


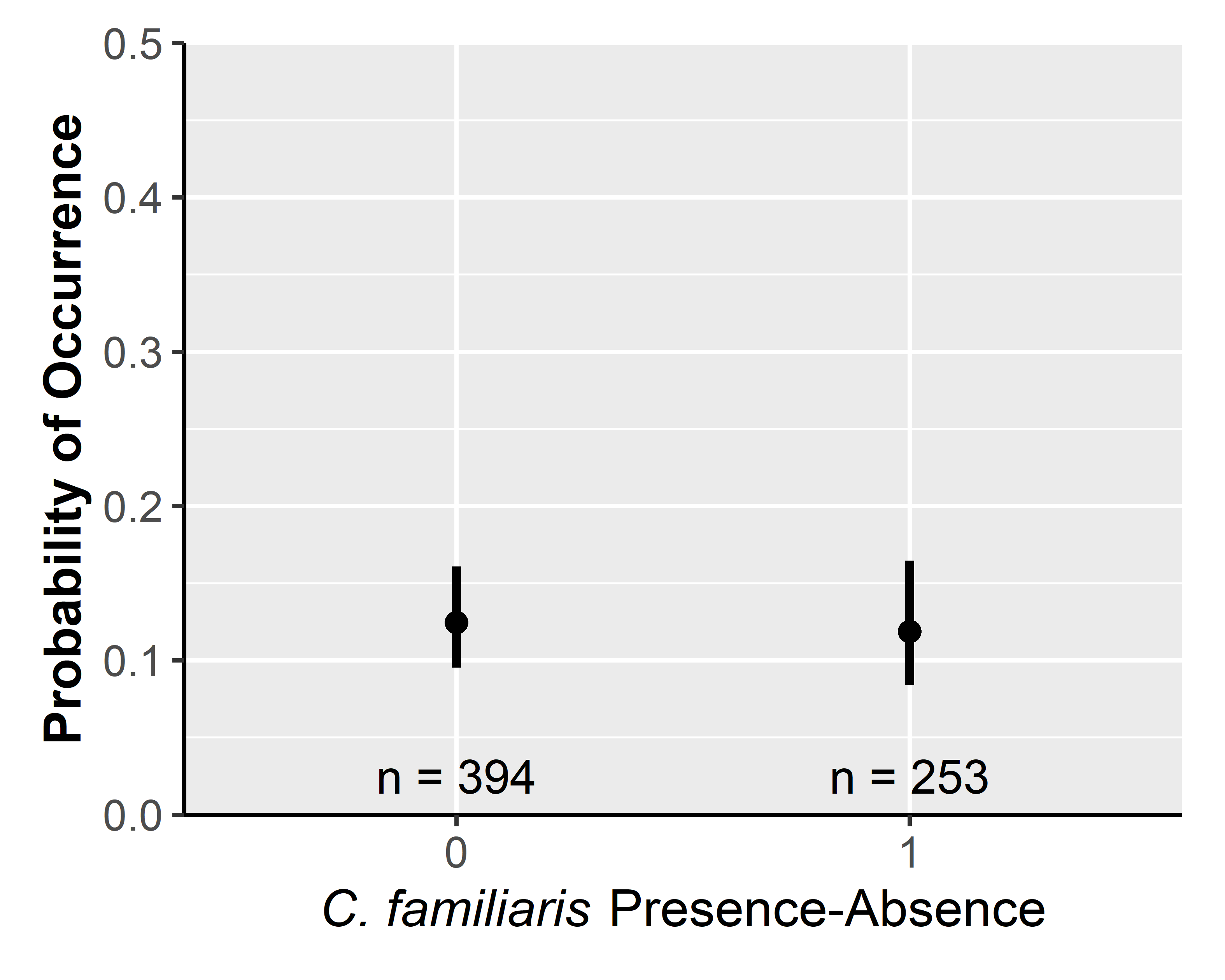


Figure S1. Predicted Probabilities of Occurrence of any ‘domestic’ prey (genera *Gallus*, *Salmo*, *Bos*, *Sus*, *Ovis*) in red fox scat samples with or without dog DNA. Line ranges represent 95% confidence interval. Sample size of each category is printed at the bottom of the panel.
